# A MEMS Fabrication Process with Thermal-Oxide Releasing Barriers and Polysilicon Sacrificial Layers for AlN Lamb-Wave Resonators to Achieve *f_s_*·*Q_m_* > 3.42 × 10^12^

**DOI:** 10.3390/mi12080892

**Published:** 2021-07-28

**Authors:** Jicong Zhao, Zheng Zhu, Haiyan Sun, Shitao Lv, Xingyu Wang, Chenguang Song

**Affiliations:** 1 School of Information Science and Technology, Nantong University, Nantong 226019, China; jczhao@ntu.edu.cn (J.Z.); 1930310034@stmail.ntu.edu.cn (Z.Z.); sun.yan@ntu.edu.cn (H.S.); 1711031169@stmail.ntu.edu.cn (S.L.); 2010310036@stmail.ntu.edu.cn (X.W.); 2 State Key Laboratories of Transducer Technology, Shanghai 200050, China; 3 School of Electrical Engineering, Nantong University, Nantong 226019, China

**Keywords:** fabrication process, aluminum nitride, lamb-wave resonators, thermal-oxide layer, motional quality factor

## Abstract

This paper presents a micro-electro-mechanical systems (MEMS) processing technology for Aluminum Nitride (AlN) Lamb-wave resonators (LWRs). Two LWRs with different frequencies of 402.1 MHz and 2.097 GHz by varying the top interdigitated (IDT) periods were designed and fabricated. To avoid the shortcomings of the uncontrollable etching of inactive areas during the releasing process and to improve the fabrication yield, a thermal oxide layer was employed below the platted polysilicon sacrificial layer, which could define the miniaturized release cavities well. In addition, the bottom Mo electrode that was manufactured had a gentle inclination angle, which could contribute to the growth of the high-quality AlN piezoelectric layer above the Mo layer and effectively prevent the device from breaking. The measured results show that the IDT-floating resonators with 12 μm and 2 μm electrode periods exhibit a motional quality factor (*Q_m_*) as high as 4382 and 1633. The series resonant frequency (*f_s_*)·*Q_m_* values can reach as high as 1.76 × 10^12^ and 3.42 × 10^12^, respectively. Furthermore, Al is more suitable as the top IDT material of the AlN LWRs than Au, and can contribute to achieving an excellent electrical performances due to the smaller density, smaller thermo-elastic damping (TED), and larger acoustic impedance difference between Al and AlN.

## 1. Introduction

In radio frequency (RF) wireless communications, micro-electro-mechanical system (MEMS) resonators have important applications, such as oscillators, filters, and duplexers, due to their small size, low power consumption, high performance, etc. [1,2]. Nowadays, surface acoustic wave (SAW) resonators and film bulk acoustic resonators (FBARs) have been dominating the RF front-end filter components market [3,4]. SAW resonators can realize multi-band integration on a single chip, which can be fabricated by using a simple and low-cost manufacturing process. However, SAW resonators can hardly achieve a high quality-factor (*Q*) value and a large power capacity. Their frequencies are limited to 3 GHz due to the low phase velocity and lithography limit [5]. Although FBARs can attain a higher frequency, higher *Q* value, and larger power capacity, it is difficult to achieve a multi-band on a single chip because its series resonant frequency (*f_s_*) and parallel resonant frequency (*f_p_*) are dominated by the thickness of the piezoelectric film [6]. In recent years, Aluminum nitride (AlN) lamb wave resonators (LWRs) have drawn widespread attention; they combine the major virtues of SAW resonators and FBARs, including a multi-band integration, high performance, complementary-metal-oxide-semiconductor (CMOS)-compatible fabrication process, etc. [7,8,9].

For AlN LWRs, an advanced manufacturing process is one of the important factors for obtaining a high performance as well as high reliability. Until now, there have mainly been three fabrication processes developed for LWRs, and the major difference between them is whether or not LWRs’ releasing cavities are formed. One process involves AlN LWRs being directly fabricated on a high-resistance silicon substrate and the silicon substrate being etched to form releasing cavities by using XeF_2_ vapor [10,11,12]. However, the isotropic etching of the silicon substrate will induce the hollowness of the inactive region and an uncontrollable releasing time, which may expand the undercut region. The extended undercut region between the resonator and the substrate causes not only an energy loss reducing the device’s *Q* value, but also the device’s fracture and an eventual performance failure. Another process involves depositing a SiO_2_ or polysilicon sacrificial layer on the Silicon wafer and etching the sacrificial layer to form an annular groove as well as define the releasing region. Then, a polysilicon or SiO_2_ film is deposited to fill the groove and flattened to form the releasing barrier by using Low Press Chemical Vapor Deposition (LPCVD) and Chemical Mechanical Polishing (CMP) processes, respectively [13,14,15]. Although the process can define the releasing cavity well, the LPCVD-deposited polysilicon or SiO_2_ film is not dense enough, which may cause releasing gas to pass through the barrier and then induce the hollowness of the inactive region. In addition, there is also a process using high-aspect-ratio SiO_2_ trenches to define the releasing region [16]. However, the release time is difficult to control due to multiple devices with different sizes on a wafer, which may cause the silicon substrate under the oxide layer to be corroded. An inactive-region etching exists for the above three processes during the releasing, which may result in the deterioration of the LWRs’ electrical performance as well as reliability.

This paper presents a MEMS fabrication process for AlN LWRs to minimize the effect from the undercut region and to obtain a high *f_s_*·*Q* value. The process defines the miniaturized release cavities by etching the silicon substrate and by thermal oxidation. The dense thermal oxide layer was used as the releasing barrier to effectively prevent the etching of the inactive region. The LPCVD polysilicon was deposited to fill the cavities and flattened by a CMP process, which acted as the releasing layer. Furthermore, the bottom Mo electrode was manufactured with a gentle inclination angle, which could contribute to the growth of the high-quality AlN piezoelectric layer and to preventing the device from breaking. The LWRs with the *f_s_* of 402.1 MHz and 2.097 GHz were fabricated with high yield in a 6-inch wafer. The measured results show that the *f_s_*·*Q_m_* values can reach up to 1.76 × 10^12^ and 3.42 × 10^12^, respectively.

## 2. Device Design

**Figure 1:**
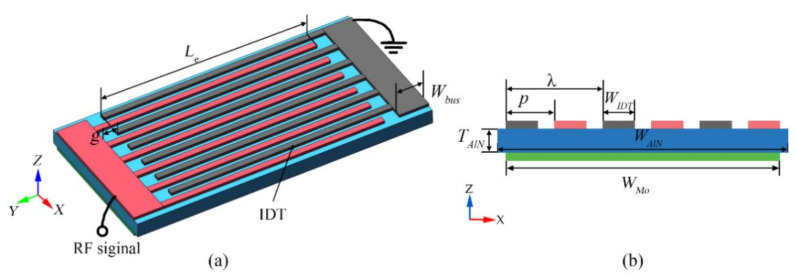
(**a**) Schematic view of the proposed Aluminum nitride (AlN) lamb wave resonators (LWRs) with floating electrodes; (**b**) Cross-section schematic of the proposed AlN LWR with a floating electrode.

Figure 1 shows the schematic view of the proposed one-port AlN LWR, which consists of a 1-μm-thick AlN film sandwiched between two metal layers. The geometrical parameters are illustrated in Figure 1, including the bottom electrode width (*W_Mo_*), AlN plate width (*W_AlN_*), AlN plate thickness (*T_AlN_*), interdigitated transducer (IDT) period (*p*), IDT width (*W_IDT_*), effective electrode length (*L_e_*), bus width (*W_bus_*), and finger-to-bus gap (*g*). The top IDT electrodes are patterned in a 200-nm-thick Al layer, alternately connected to the ground and RF signals. The bottom Mo electrode with a thickness of 200 nm is patterned to a rectangular plate, which is set as electrically floating. The top IDTs and bottom electrode are combined to actuate Lamb-wave modes in the AlN film, and the IDTs are also used to sense resonant signals. The frequency of LWRs is mainly dependent on the *p* of IDTs, which can be expressed in Equation (1) [17,18]:

(1) $$f=\frac{v}{\lambda }=\frac{v}{2p}$$

where *v* is the Lamb-wave phase velocity, and *λ* is the wavelength shown in Figure 1b.

**Figure 2:**
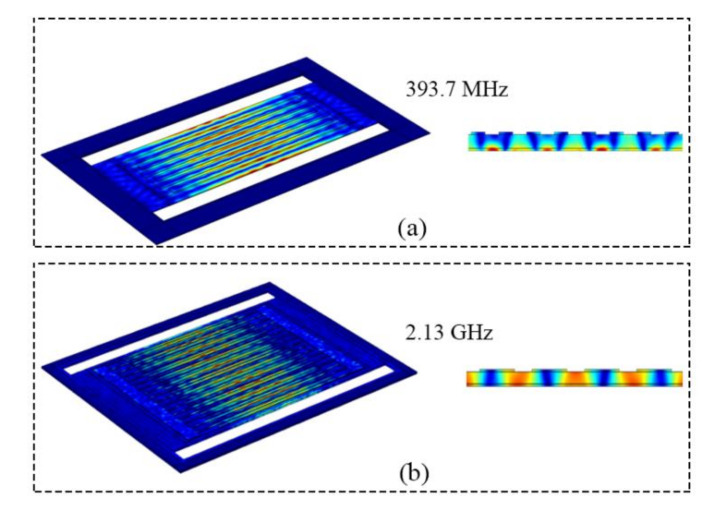
Simulated displacement-field distributions of the two LWRs with the IDT periods of (**a**) 12 μm and (**b**) 2 μm.

For LWRs, ideal harmonic conditions are met when the IDTs’ central regions are located at the potential maximums and displacement amplitude minimums. In this work, two LWRs with different IDT *p* values of 12 μm and 2 μm were designed, and their calculated resonant frequencies were 393.7 MHz and 2.13 GHz by using COMSOL Multiphysics V4.3a software, respectively. Figure 2 shows the simulated resonant modes of the two LWRs. It is clearly seen that the maximum displacements are all located at the centers and edges of the AlN plates and that the desired Lamb-wave modes are strongly excited. The key geometric parameters of the two LWRs were designed as shown in Table 1. The IDT numbers (*n*) of the two LWRs with the IDT *p* of 12 μm and 2 μm were designed as 6 and 34, respectively. Additionally, the AlN plates are one IDT period wider than the bottom Mo electrode on each side. On the one hand, the design can avoid the exposure of the Mo electrode at the reflection boundary due to lithography deviations, which may induce the undesired etching of the Mo electrode during XeF_2_ releasing. On the other hand, the widening of one IDT period can ensure that the Lamb wave is reflected in the region of maximum displacement, which can maintain spectral purity and a high *Q* value.

## 3. Fabrication Process and Results

**Figure 3:**
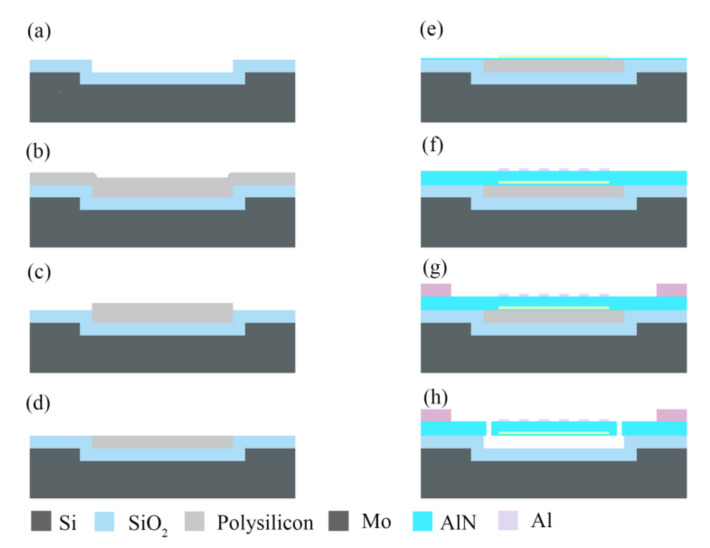
The fabrication process of the AlN LWRs: (**a**) silicon etching and thermal oxidation; (**b**) polysilicon deposition; (**c**) polysilicon etching; (**d**) polysilicon CMP; (**e**) AlN seed layer and Mo deposition, then Mo etching; (**f**) AlN deposition, and top IDTs formation; (**g**) Al Pads formation; (**h**) AlN etching and XeF_2_ releasing.

The AlN LWRs were microfabricated on a 6-inch silicon wafer with a resistivity of 10,000 Ω·cm. The designed fabrication process based on six-step lithography is shown in Figure 3. The fabrication of devices started by defining the release cavities with a 1-μm depth by reactive ion etching (RIE). Then, a 1-μm-thick SiO_2_ film was formed on the wafer by using thermal oxidation, as shown in Figure 3a. The SiO_2_ film acts as a release barrier during the XeF_2_-based releasing, avoiding the performance attenuation of the LWRs induced by uncontrollable undercut regions. Next, a polysilicon layer with a 1.5-μm thickness was deposited by using the LPCVD process to refill the release cavities, as shown in Figure 3b. Then, the polysilicon outside the cavities was removed by RIE, as shown in Figure 3c, which could contribute to a quick flattening of the wafer as well as to avoiding the problem of cavity depression or over-polishing of polysilicon. The following CMP process was adopted to grind the remaining polysilicon to be flush with SiO_2_ outside the cavities, as shown in Figure 3d. In this step, the step height at the boundaries of the release regions should be below 45 nm, which is beneficial to reducing the stress concentration of the AlN film at the boundaries.

**Figure 4:**
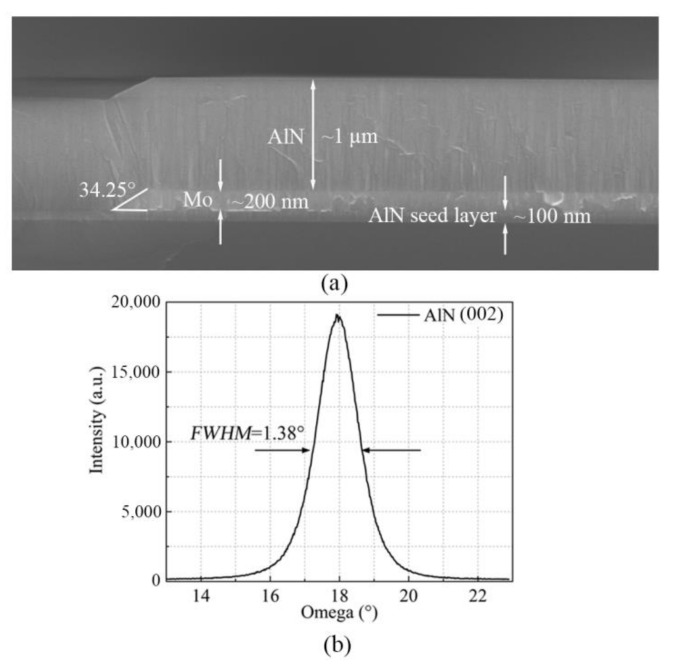
(**a**) SEM images of the cross section of the Mo sidewall; (**b**) XRD rocking curve of the 1-μm-thick AlN (002) layer.

After the fabrication of the release cavities and sacrificial layer, a AlN seed layer with a thickness of 100 nm was deposited by magnetron sputtering (MS) (Figure 3e), which could prevent Mo corrosion by XeF_2_. With the AlN seed layer, the bottom Mo electrode can achieve a better (110) crystalline orientation, which then contributes to the (002) orientation growth of the AlN film. Then, a 200-nm-thick Mo layer was deposited and patterned to form the bottom Mo electrodes. In this step, the Mo electrode edges should be at a shear angle of 10–45° to prevent cracking in the following-deposited AlN layer. Before RIE, the photoresist mask was baked to be slanted at 150 °C for 30 min. The etching selection ratio of Mo was reduced by optimizing the flow rates of etching gases, which could effectively transfer the inclination morphology of the photoresist mask to the Mo electrodes. In Figure 4a, the scanning electron microscope (SEM) image shows that the fabricated Mo electrodes have a shear angle of 34.25°. Next, the AlN piezoelectric layer with a thickness of 1 μm was deposited by MS, and an X-ray diffraction (XRD) rocking curve was measured to study the AlN crystallinity, which corresponded to the c-axis-oriented AlN (002) crystal structure. As shown in Figure 4b, the full width at half maximum (*FWHM*) value is 1.38°, indicating that the AlN piezoelectric layer has an excellent crystalline quality.

**Figure 5:**
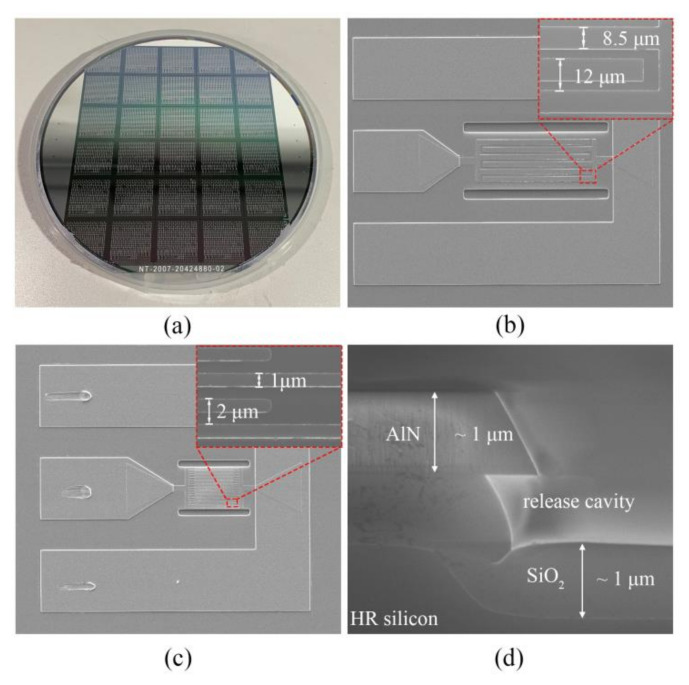
(**a**) The fabricated 6-inch wafer; the fabricated AlN LWRs with the IDT periods of (**b**) 12 μm and (**c**) 2 μm; (**d**) cross-sectional view of the device after release.

Ti (20 nm)/Al (180 nm) layers and then Ti (20 nm)/Al (1 μm) layers were deposited on the AlN piezoelectric layer and patterned to serve as the top IDTs and electrical Pads by a lift-off process, respectively. Next, AlN piezoelectric (1 μm) and AlN seed (100 nm) layers were etched by RIE, which defined the desired resonant structures as well as release tapping. Finally, the fabricated 6-inch wafer with LWRs were released by XeF_2_ gas, and the wafer is shown in Figure 5a. The SEM images of the fabricated LWRs with the IDT periods of 12 μm and 2 μm are shown in Figure 5b,c. Figure 5d is a cross-sectional view of the device after release, from which the thermal oxide layer and the release cavity can clearly be seen.

## 4. Measurement and Discussions

A Keysight’s N5244A vector network analyzer (VNA) and a Cascade SA8 probe station were used to measure the transmission characteristics of the fabricated LWRs in air, and the signal power was set as 0 dBm (1 mW). Before testing the frequency responses, a standard short-load-open-through (SLOT) calibration was performed. The LWRs designed in this paper were all one-port devices, so their reflection scattering parameters *S*_11_ were recorded by the VNA. The admittance *Y*_11_ can be given in terms of the measured *S*_11_ in Equation (2):

(2) $$Y_{11}=\frac{1}{Z_{0}}\frac{1-S_{11}}{1+S_{11}}$$

where *Z*_0_ is set as 50 Ω, which is the source or load impedance of the VNA. The admittance *Y*_11_(dB) can be obtained via Equation (3):

(3) $$Y_{11}(\mathrm{dB})=20\log_{10}\left|Y_{11}\right|=20\log_{10}(\sqrt{real{\left(Y_{11}\right)}^{2}+imag{\left(Y_{11}\right)}^{2}})$$

**Figure 6:**
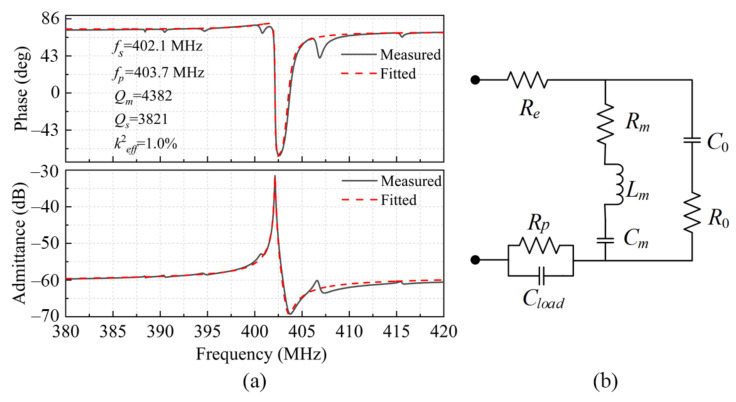
(**a**) The measured and fitted admittance diagram and phase diagram of the LWR with the IDT period of 12 μm; (**b**) the MBVD model used to extract the LWRs’ equivalent electrical parameters.

Figure 6a shows the measured admittance *Y*_11_(dB) and phase of the LWR with the IDT period of 12 μm. The measured series resonant frequency *f_s_* is 402.1 MHz, which is in good agreement with the simulation result of 393.7 MHz. The small deviation may have been caused by the fabrication errors of the geometric dimensions and by the slight discrepancy of AlN Young’s modulus between the simulation and the actual fabrication. The effective electromechanical coupling coefficient (*k*^2^*_eff_*) can be extracted as 1% via Equation (4) [19]:

(4) $$k^{2}{}_{eff}=\frac{\pi^{2}}{4}\frac{\left(f_{p}-f_{s}\right)}{f_{p}}$$

In order to accurately describe the fundamental mode of the resonant, a Modified Butterworth–van Dyke (MBVD) circuit model was used to extract the device properties, as shown in Figure 6b. The mechanical resonance is described by a motional branch in parallel with the series connected capacitor *C*_0_ and resistor *R*_0_. In the motional branch, the series connected motional resistor *R_m_*, capacitor *C_m_*, and inductor *L_m_* have a dominant influence on the series resonance, corresponding to the damping, stiffness, and mass of the resonator, respectively. The *C*_0_ and *R*_0_ mainly determine the parallel resonance, representing the device’s static capacitance and the parasitic resistance in the substrate, respectively [20]. The resistor *R_e_* denotes the resistance of the IDTs and Pads, while the resistor *R_p_* and *C_load_* are the resistance and the capacitance of the load [21]. The optimized MBVD model fits the measurement data with a high accuracy, as shown in Figure 6a, and the extracted equivalent electrical parameters are given in Table 2. The motional quality factor (*Q_m_*) represents the loss level of the device’s mechanical energy, which can be calculated by using the MBVD-extracted lumped element parameters through Equation (5) [22]. The series resonance quality factor (*Q_s_*) can be extracted via Equation (6):

(5) $$Q_{m}=\frac{2\pi f_{s}L_{m}}{R_{m}}$$

(6) $$Q_{s}=\frac{1}{\omega_{s}(R_{m}+R_{e}+R_{p})C_{m}}$$

where *ω_s_* is the angular resonant frequency (*ω_s_* = 2π*f_s_*).

For the LWR with the IDT period of 12 μm, the calculated *Q_m_* and *Q_s_* are as high as 4382 and 3821, respectively, thanks to the advanced manufacturing technology as well as the optimized device structure. The extremely high *Q_m_* and *Q_s_* are mainly due to the well-defined miniaturized release cavity as well as the high-quality deposition of the c-axis-oriented AlN piezoelectric layer.

**Figure 7:**
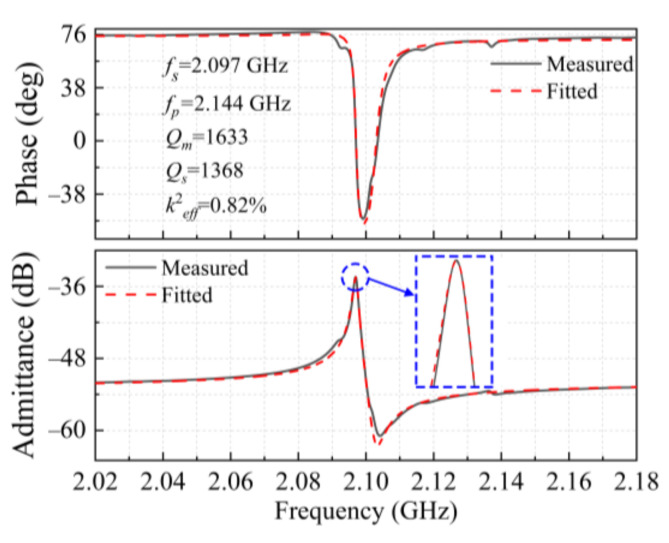
The measured and fitted admittance diagram and phase diagram for the LWR with the IDT period of 2 μm.

For the LWR with the IDT period of 2 μm, its admittance *Y*_11_(dB) and phase were calculated from the measured reflection scattering parameters *S*_11_, and the MBVD circuit model was used to fit the measured curves, as shown in Figure 7. Its extracted equivalent electrical parameters are shown in Table 2. The measured series resonant frequency *f_s_* is 2.097 GHz, which is highly consistent with the simulated value of 2.13 GHz. The LWR can obtain a *k*^2^*_eff_* of 0.82%, but its *Q_s_* and *Q_m_* values of 1368 and 1633 are well below those of the LWR with the IDT period of 12 μm. The relationship between the device’s *Q* value and various losses can be expressed as Equation (7) [23]:

(7) $$\frac{1}{Q}=\frac{1}{Q_{\mathrm{anchor}}}+\frac{1}{Q_{\mathrm{TED}}}+\frac{1}{Q_{\mathrm{material}}}+\frac{1}{Q_{\mathrm{interface}}}+\frac{1}{Q_{p-p}}+\frac{1}{Q_{\mathrm{other}}}$$

Aside from intrinsic material limitations (*Q_material_*), commonly encountered energy loss mechanisms for MEMS resonators include anchor loss (*Q_anchor_*), interface loss (*Q_interface_*), thermoelastic damping (TED) loss (*Q_TED_*), and the loss caused by phonon–phonon interaction (*Q_p-p_*). When the resonator approaches the GHz regime, the *Q* value is mainly limited by a combination of the TED loss and local phonon–phonon scattering. In this case, the LWR’s *Q_m_* value decreases from 4382 to 1633 with its frequency increasing from 402.1 MHz to 2.097 GHz.

**Figure 8:**
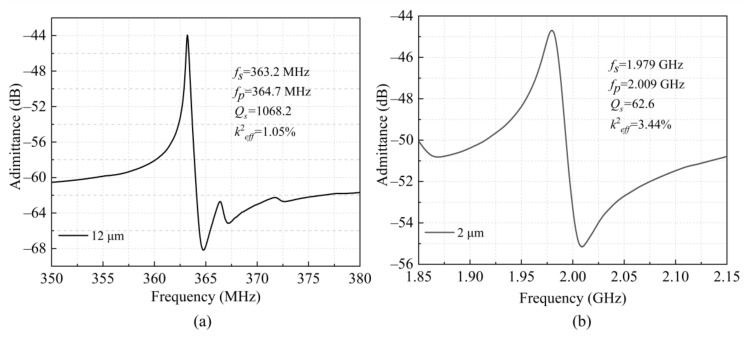
Admittance diagrams of LWRs using Au as the top electrode material with the IDT electrode period of: (**a**) 12 μm and (**b**) 2 μm.

In addition, the IDT electrodes material also has a significant influence on the LWRs’ performance. The above-analyzed LWRs use Al as the IDT material. In this work, we also fabricated LWRs with Au IDTs, which had low electrical impedances similar to that of Al IDTs. Figure 8 shows the admittance curves of the LWRs with the Au IDT periods of 12 μm and 2 μm. Compared to the LWRs with the Al IDTs, their series resonant frequencies decrease from 402.1 MHz and 2.097 GHz to 363.2 MHz and 1.979 GHz, respectively, which is mainly induced by a higher density of Au. The higher density of Au will result in a larger mass-load effect and a lower phase velocity, which can both contribute to the frequency reduction. Besides, there is a serious degradation to the *Q_s_* values of the LWRs with Au IDTs. In particular, the LWR with the IDT period of 2 μm has a very small *Q_s_* value of 62.6. The *Q_s_* attenuation is mainly caused by the following two factors: the TED loss of the Au IDTs is much higher than that of the Al IDTs; the better acoustic impedance matching between AlN and Au will induce obvious acoustic-energy dissipation. The mass density and acoustic impedance of different materials are listed in Table 3 [24].

In this work, the LWRs with series resonant frequencies of 402.1 MHz and 2.097 GHz were designed and fabricated, and their *f_s_·Q_s_* values could reach 1.53 × 10^12^ and 2.86 × 10^12^, respectively. A high Figure of Merit (*FOM* = *k*^2^*_eff_ × Q_s_*) of 43.82 and 11.21 was obtained, which was comparable with that of reported AlN LWRs [10,25,26,27], as shown in Table 4. The experimental results demonstrate that the proposed fabrication process can be capable of producing LWRs with a high *f_s_·Q_s_* and moderate *FOM*.

## 5. Conclusions

In this paper, we reported a fabrication process for AlN LWRs. The miniaturized release cavities were strictly defined by etching a high-resistance silicon substrate. A thermal oxidation layer was used as the releasing barrier to effectively prevent the etching of the inactive region by XeF_2_, which could effectively improve the performance and the yield of the device. Moreover, the bottom Mo electrode had a moderate shear angle of 34.25°, which contributed to a high-quality deposition of AlN. The measured *FWHM* value was 1.38°, indicating that the piezoelectric layer had an excellent crystalline quality.

For the LWRs with IDT periods of 12 μm and 2 μm, their measured *f_s_* and *Q_s_* were 402.1 MHz and 2.097 GHz, 3821 and 1368, respectively. The *FOM* values were obtained by using LWRs. Based on the measured frequency responses, an optimized MBVD circuit model was used to extract LWRs’ equivalent electrical parameters. The *Q_m_* values of the LWRs with IDT periods of 12 μm and 2 μm were calculated to be as high as 4382 and 1633, respectively. Compared to Au, Al is more suitable as an IDT material for LWRs due to its lower TED loss and higher degree of acoustic impedance mismatch with AlN.

## Figures and Tables

**Table 1:** Geometric dimensions of the designed AlN LWRs.

Top IDT Period (*p*)	12 μm	2 μm
IDT numbers (*n*)	6	34
Bottom electrode width (*W_Mo_*)	72 μm	68 μm
AlN plate width (*W_AlN_*)	96 μm	72 μm
IDT width (*W_IDT_*)	8.5 μm	1 μm
Effective electrode length *(L_e_*)	192 μm	60 μm
Finger-to-bus gap (*g*)	6 μm	9 μm
Bus width (*W_bus_*)	7 μm	6 μm

**Table 2:** Electro-mechanical parameters extracted from the measured frequency responses of AlN LWRs.

IDT Period	12 μm	2 μm
*R*_0_ (Ω)	287.25	99.21
*C*_0_ (fF)	432.1	188.08
*C*_load_ (fF)	9.23	1.18
*R_m_* (Ω)	31.95	45.43
*C_m_* (fF)	2.82	1.03
*L_m_* (μH)	55.5	5.58
*R_e_* (Ω)	4.92	5.73
*R_p_* (Ω)	1.24	2.98

**Table 3:** Mass densities and acoustic impedances of AlN, Al, and Au.

	AlN	Al	Au
Mass density (kg/m^3^)	3260	2700	19,300
Acoustic impedance (C/m^2^)	1061	436	1162

**Table 4:** The key performance parameters of the fabricated LWRs, compared to a variety of reported LWRs.

Electrical Properties	Ref. [25]	Ref. [10]	Ref. [26]	Ref. [27]	This Work	This Work
*f_s_* (MHz)	243	865	1380	2740	402.1	2097
*Q_s_*	4050	2968	2181	540	4382	1368
*k* ^2^ *_eff_*	1.75%	0.27%	0.71%	5.64%	1.0%	0.82%
*f_s_*·*Q_s_*	9.84 × 10^11^	2.57 × 10^12^	3.01 × 10^12^	1.48 × 10^12^	1.53 × 10^12^	2.86 × 10^12^
*FOM*	7.03	8.01	15.49	30.5	43.82	11.21
